# “She’s loved me at all sizes”: the role of the human-animal bond in eating disorder experiences and treatment

**DOI:** 10.1186/s40337-026-01643-5

**Published:** 2026-05-13

**Authors:** Annalyse Ellis, Marie-Christine Opitz, Steve Loughnan, Roxanne D. Hawkins

**Affiliations:** 1 Department of Psychology, School of Philosophy, Psychology, and Language Sciences, University of Edinburgh, 3 Charles St, Edinburgh, EH8 9AD UK; 2 Department of Clinical and Health Psychology, School of Health in Social Science, University of Edinburgh, Old Medical School, Elsie Inglis Quadrangle, Teviot Pl, Edinburgh, EH8 9AG UK

**Keywords:** Eating disorders, Pet ownership, Affect regulation, Personal recovery, Treatment motivation, Eating pattern stabilization, Compensatory exercise

## Abstract

**Background:**

The mental well-being benefits and challenges of pet ownership have been explored in many populations, including clinical populations such as individuals with depression, anxiety, and posttraumatic stress disorder. Critically, there are currently no published studies focusing specifically on pet ownership for individuals with eating disorders. The present work aimed to explore pet ownership experiences of adults experiencing eating disorders.

**Methods:**

Semi-structured interviews were conducted with 17 cat- and dog-owning British adults (eight cat owners, six dog owners, three participants with both cats and dogs) with diagnosed or suspected eating disorders. Reflexive thematic analysis was utilized to identify two overarching themes and seven subthemes relating to the role of the human-pet bond in eating disorder symptoms and treatment.

**Results:**

Data-driven themes include personal recovery experiences, the dual role of pets in eating behaviors, exercise experiences, pets’ influence on cognitions, body image perceptions, and self-identity, as well as pets’ roles in personal recovery motivations. Furthermore, pets were found to impact treatment experiences, represented by themes that discuss pets as catalysts for change and partners during treatment, while also creating challenges around separation during treatment.

**Conclusions:**

The present study found that pets can be largely beneficial for individuals with eating disorders, especially for those in later stages of personal recovery; however, pets can also create challenges, especially for individuals in earlier stages of personal recovery. The present work also provides a foundation for future work related to pet ownership for individuals with eating disorders, such as further exploration on incorporating pets into eating disorder treatment, how to utilize the human-pet relationship to support symptom reduction, and how to mitigate challenges posed by pets related to treatment, with the results having key relevance for the development of clinical interventions for this population.

## Abstract (plain language summary)

The study examined the relationships that people with eating disorders have with their pets. Semi-structured interviews were conducted with 17 participants who owned cats or dogs. Two overarching themes and seven subthemes were identified. These themes included the role pets play in personal recovery experiences and personal recovery motivation, the dual function of pets in eating behaviors and exercise experiences, how pets influence thought patterns, perceptions of body image, and self-identity, and how pets provide motivation and support, but also challenges related to treatment. The findings of this study provide a better understanding of the support, but also challenges, related to pet ownership for people with eating disorders, and provide a foundation for future research in this area. Future work building on the present study may focus on incorporating pets into eating disorder treatment, how to utilize pets to support symptom reduction, and how to mitigate challenges posed by pets related to treatment.

## Background

Eating disorders (ED) are both complex and prevalent mental health concerns, with high mortality and relapse rates [37]. There is abundant research related to the role of companion animals (hereafter “pets”) in mental ill-health, with existing work yielding variable results related to the “pet effect” (e.g., the well-being benefits derived from pet ownership; for reviews, see [9, 54, 58]. Existing work related to pet ownership and mental well-being has been conducted within a broad range of populations, including populations with a variety of mental health concerns [5, 23, 34, 61]. However, there are currently no published studies focusing solely on the experiences of pet-owning individuals with EDs. This study will investigate the ways in which pets, specifically cats and dogs, impact individuals with EDs.

The definition of ED recovery is complex, with clinical definitions of recovery encompassing behavioral, cognitive, and psychosocial domains, focused on symptom improvement, decreased ED-related thought patterns, and increased social connection [24, 64]. However, the definition of personal recovery expands this definition of recovery to include broader factors such as overall quality of life, sense of identity, and internal transformation [64]. The present work will focus on this more holistic definition of personal recovery. There are many key aspects of ED personal recovery, including stabilization of eating patterns, building a healthy relationship with exercise, coping with negative moods, reducing self-criticism related to shape and weight and, importantly, increasing relational security [2, 27, 30, 40, 65]. Low levels of positive social support and dissatisfaction with social support are risk factors for disordered eating [29, 44], while positive social support and relational security often play a key role in ED treatment and recovery (for a review, see [42]). In addition to social support and relational security, social and relational identity is an important factor in ED illness and recovery [24, 49]. Despite existing literature pointing to the crucial role of the support system for individuals with EDs, and prior research indicating that the bonds people experience with their pets are often highly important, close, and supportive [3, 6]; the roles of pets in the lives of individuals with EDs remains largely unexplored.

The human-pet relationship is rich and complex, with pets providing support, closeness, and companionship [50]. The “pet effect” has been widely studied, with some studies indicating that pet ownership is linked to better mental well-being, such as lower levels of depression and anxiety [5, 11, 26, 45, 56]. However, other work has found that pet owners experience unique stressors related to their pets, and higher levels of depression and anxiety than non-pet owners [4, 12, 21, 31, 52]. The variability in results indicates that the human-pet relationship is highly complex. Existing qualitative studies provide much needed nuance related to the human-animal interactions field’s understanding of these relationships, highlighting the complex interplay between the human-pet relationship and mental well-being. These studies indicate that while pets often provide benefits related to mental well-being, they also can create emotional strain and stress in certain situations [33, 59]. Thus, the same pet may provide both well-being benefits and challenges to an individual depending on the current context. Due to the nuance and depth of information provided by qualitative studies, which demonstrate that the human-pet relationship fluctuates in terms of benefits provided and challenges posed; the benefits of lived experience; and due to the shortage of existing research related to pet ownership for individuals with EDs, we chose to conduct the present work qualitatively.

There is little existing literature regarding the role of pets in ED recovery. One prior study, which focused on factors impacting the quality of life for individuals with EDs, found that pets can contribute positively to quality of life [19]. Additionally, there are two studies to our knowledge that have explored the role of pets in the mental well-being of individuals with EDs: one study which included one participant with an ED among a broader sample of emerging adults with mental health concerns, and another study which was an unpublished dissertation. The first qualitative study found that for a participant who had experienced eating difficulties at regular mealtimes, eating together at regular mealtimes with her dog was helpful for stabilizing eating patterns [34]. The second study, an unpublished dissertation [55], qualitatively explored the experiences of seven pet owners with eating disorders and found that pets were able to offer participants a sense of connection, companionship, love, and purpose, and helped participants become more mindful. As these three studies comprise the full extent of the existing literature regarding pet ownership for individuals with EDs, it is clear that significant gaps in knowledge remain.

There are many ways in which pets could potentially impact individuals with EDs, including several key aspects associated with ED recovery, such as eating patterns, compensatory exercise, affect regulation, self-identity, recovery motivation, and treatment engagement. Because it is recommended that both dogs and cats should be fed at least two times per day [13, 57], eating some meals with pets could be a mechanism for stabilizing eating patterns, as seen in Hawkins et al., [34]. Pet owners, particularly dog owners, often engage in regular exercise (e.g., walks) with their pets which can be beneficial for health and well-being [39, 46], but could also potentially exacerbate compensatory exercise behaviors for those with EDs. Furthermore, individuals with EDs often experience high levels of affect dysregulation [43, 51]. Importantly, pet ownership and interacting with pets is associated with more positive affect and less negative affect [35, 38, 62], and pets can provide comfort, alleviate stress, and reduce the onset of panic attacks [9, 32, 33, 48]. Finally, individuals with EDs often experience a restricted sense of self-identity, over-evaluating shape and weight to the detriment of engaging in other areas of life, with this restricted sense of self-identity being linked with a higher likelihood of ED behaviors [27, 28, 60]. As pet owners commonly integrate pet ownership into their self-concept, and pets can help enhance a sense of self-identity [8, 23], pet ownership has the potential to positively impact people with eating disorders. In addition, pets have been shown to provide support and a reason to recover in other populations such as individuals in substance use disorder recovery, older adults after a stroke, and general mental ill-health recovery [34, 36, 41]. These findings suggest that pet ownership could play an important role in addressing general and specific mental health concerns in individuals with eating disorders.

Based on the existing literature related to pets and mental well-being, as well as the limited existing work regarding pets and individuals with EDs, it is likely that pets impact the lives of individuals with EDs in a variety of ways. Qualitative research provides much needed nuance regarding people’s complex relationships with pets, and therefore the present work is undertaken drawing on qualitative methodologies. No hypotheses were made regarding how pets may impact individuals with EDs, however, this study aimed to further explore how pets may influence patterns of eating as well as compensatory exercise, affect regulation, self-identity, motivation for recovery, and impact on ED treatment, as we believe that these areas reflect key gaps in the field’s knowledge.

## Method

### Design and participants

This study aimed to better understand the experiences of pet-owning individuals with EDs and utilized a qualitative approach in which semi-structured interviews were conducted online via Microsoft Teams. Inclusion criteria consisted of the following: (1) living in the United Kingdom; (2) owning at least one dog or cat; (3) having been diagnosed with or self-identifying as having an eating disorder or experiencing symptoms of an eating disorder; and (4) fluent in English. The age range of participants was 18–35 (M = 26.1). Eight participants were cat owners, six were dog owners, and three participants had both cats and dogs. Additionally, four participants had other types of pets including fish (*n* = 1), insects (*n* = 1), rabbits (*n* = 2), and birds (*n* = 1). However, due to our research focus on cat and dog ownership, as well as the small number of participants with pets of other species, we have chosen not to include an analysis of these pet ownership experiences. Fifteen participants were living with their pets at the time of the interview, while two were not. Fifteen participants reported a diagnosis of anorexia nervosa, three participants reported a diagnosis of avoidant restrictive food intake disorder, one participant reported a diagnosis of atypical anorexia, one participant reported a diagnosis of orthorexia, and one participant reported a diagnosis of other specified feeding or eating disorder. Participants were also asked about other mental health diagnoses and neurodivergence, and participants reported diagnoses or suspected diagnoses of depression (*n* = 11), autism (*n* = 10), anxiety (*n* = 9), attention-deficit/hyperactivity disorder (*n* = 6), posttraumatic stress disorder (*n* = 4), obsessive compulsive disorder (*n* = 4), bipolar disorder (*n* = 2), substance use disorder (*n* = 2), separation anxiety disorder of childhood (*n* = 1), social anxiety disorder (*n* = 1), borderline personality disorder (*n* = 1), emotionally unstable personality disorder (*n* = 1), and general neurodivergence (*n* = 1). Participant names have been changed to pseudonyms throughout.

### Procedure

Participants (*N* = 17) were recruited via convenience and snowball sampling where study flyers and information were shared on Reddit subreddits focused on eating disorders. Individuals interested in taking part in the study could register their interest by scanning a QR code within the study flyer or clicking a link to the study sign-up page. The sign-up page provided detailed study information, and prospective participants gave informed consent. Prospective participants also provided their names, contact information, availability, and their eating disorder diagnoses or suspected diagnoses. Prior to official enrollment in the study, participants met with a researcher briefly online (less than 10 min) to ensure eligibility criteria were met and to discuss any questions. Participants who met eligibility criteria and were still interested in participating in the study were then scheduled for an interview.

All interviews were one-on-one with a researcher, and took place online. Participants were reminded of their rights, as well as the study’s aim, and had the opportunity to ask questions about the study at the beginning of the interview. Participants were also asked to provide an emergency contact due to the sensitive nature of the interviews. Interviews were recorded and transcribed; recordings were deleted upon the completion of transcription. The interviews lasted between 32 and 66 min, with the average length of interview being 46 min. At the end of the interview, participants were given the opportunity to ask questions about the study and were reminded that they could email any further questions should they arise and should they wish to do so. Participants were also asked if they were interested in learning about the study results, and all participants expressed interest. After the interviews, a member of the research team emailed participants thanking them for their participation, with a £15 shopping voucher for their participation and debriefing information with relevant mental health resources.

### Data analysis

Data were analyzed using reflexive thematic analysis, a data analysis methodology with six phases: (1) familiarization with the data, (2) generation of initial codes, (3) searching for themes, (4) reviewing themes, (5) defining and naming themes, and (6) writing up the thematic analysis [7]. These steps were undertaken collaboratively, with all members of the research team reaching agreement on the final themes.

## Results

Two overarching themes and seven subthemes (four in Theme 1 and three in Theme 2) were identified through reflexive thematic analysis. The first theme focuses on the emotional, cognitive, and behavioral impact of pets, specifically including the dual role of pets on eating and exercise behaviors, the influence of pets on self-identity and thought patterns, and recovery motivation. The second theme focused on the role of pets in treatment, specifically focusing on pets as a catalyst for treatment engagement, the supportive roles of pets related to treatment, and challenges associated with pets and treatment engagement.

### Theme 1: the emotional, cognitive, and behavioral impact of pets

*Summary*: This theme captures the complex beneficial and detrimental roles that companion animals play regarding eating disorder-specific emotions, behaviors, and thoughts. These roles seemed to change across the recovery journey, with pets contributing to more unhealthy patterns at the start of participants’ recovery journeys and then becoming more of a support and catalyst for positive change as participants were in later stages of recovery. Participants specifically discussed ways in which their pets had impacted their compensatory exercise behaviors, eating behaviors, thought patterns, and recovery motivation. 

#### Subtheme 1A: pets as comfort and challenge - the dual role in eating behaviors

Many participants felt that their pets had played a role in their eating patterns. However, whether pets provided benefits or created challenges varied based on recovery stage for many participants, with participants in earlier stages of recovery generally experiencing more challenges.

Challenges related to pets and eating behaviors included sharing food with pets as a way to reduce one’s own food intake, barriers to following meal plan routines posed by prioritizing their pets’ needs, and sensory concerns posed by pet ownership leading to food aversion. Firstly, three participants reported that in the past, prior to recovery, they would share food with their pets (both cats and dogs) to avoid eating the food themselves:

*“When I was slightly younger*,* I probably might have used him [cat] as a sort of like*,* maybe if*,* like*,* I had a bit of food that I didn’t want to eat*,* I might have given it to him.” (Anna*,* cat owner)*.

*“When I was like going through a bad time*,* it’s quite easy to feed her [dog] food on my plate*,* that’s not like noticeable by other people.” (Gabriella*,* dog owner)*.

*“I’ve been known to sort of share my food with her [dog]*,* so it might look like I’m eating X amount*,* but she’s getting some of it.” (Laura*,* dog owner)*.

These quotes reflect how participants’ relationships and interactions with their pets may have facilitated the opportunity to mask and conceal disordered behaviors around eating while unwell.

Furthermore, Ben reported that his dog’s appointments posed barriers to following his meal plan leading to missing his own meals:

*“If he’s [dog] got like his hydrotherapy appointments or physiotherapy appointments around like lunchtime or early morning*,* I’ve used that as an excuse not to have a meal.” (Ben*,* dog owner)*.

This participant found that his obligations related to pet ownership fueled disordered and restrictive eating patterns.

Additionally, Mia found that her cat would sometimes sniff her food, which she found disruptive to her eating:

*“I went through a phase where I was mainly eating like in our front room on the sofa. So she [cat] would like climb up there and she would be sniffing around my plate. And I found that really like challenging because I found myself getting annoyed with her.” (Mia*,* cat owner)*.

This participant also reported experiencing Obsessive Compulsive Disorder (OCD) and found her cat’s sniffing to be particularly challenging for this reason, highlighting the complex interplay between eating disorders, other mental health concerns, and pet ownership.

However, participants also identified several ways in which pets had been helpful in terms of eating patterns, such as emotion regulation, distraction, routines helping to stabilize eating patterns, and pets modeling healthy relationships with food. Relating to emotion regulation, pets were able to help soothe participants both prior to a meal, and when experiencing unpleasant emotions after a meal, preventing negative emotions (e.g., anxiety) and unhealthy behaviors (e.g., purging). For example, Kelsey noted that her cats helped her generally feel less anxious, enabling her to feel calm enough to eat at mealtimes.

*“They [cats] make me feel calmer and less anxious*,* and anxiety plays a big part in my eating disorder and being able to feel settled enough to eat.” (Kelsey*,* owner of two cats)*.

This quote highlights that not only can pets help with distress after a meal, but also with general emotional well-being, which can be helpful in maintaining recovery-oriented behaviors.

A pet’s physical presence during and after mealtimes was enough to regulate emotions, preventing unhealthy emotions such as distress, and unhealthy behaviors, as described by Ben and Mia:

*“Once I’ve eaten*,* I make sure I sit down and that he’s [dog] with me*,* sort of to keep me calm and to stop me sort of making myself sick or doing like exercising afterwards.” (Ben*,* dog owner)*.

*“I might feel a bit bad about eating something and if she’s [cat] just like lying at the top of the stairs and I can like lie next to her and like. I find it kind of calming just to be in her presence.” (Mia*,* cat owner)*.

Ben also found that being able to do physiotherapy for his dog or take a walk with his dog was helpful as a way to distract from distress after eating:

*“After*,* like I’ve had a snack*,* I usually do it [dog’s physiotherapy] either before or after a walk*,* so that keeps me distracted as well. Or like after I’ve had breakfast*,* I do his [dog] physio.” (Ben*,* dog owner)*.

This quote highlights that caring for a pet through regular routines can offer a meaningful distraction from distress, thereby helping with recovery-oriented behaviors.

Several participants also reported eating meals with pets or utilizing their pets’ schedules to stabilize their own eating patterns. A key mechanism was pets providing an important sense of routine and structure around meals which provided a ‘*reason to eat’* or a ‘reminder to eat’ and encouraged healthy eating habits:

*“Because he [dog] eats at 5:00*,* it means I have to eat at 5:00. So it sort of gives me a time that I have to eat at least one time a day*,* and I’ve now sort of give started giving him a lunch as well*,* so that gives me a reason to eat at lunchtime.” (Ben*,* dog owner)*.

*“All my pets have provided me like with a bit of daily structure*,* which I find really helpful. If I know that I have to do that for my animal*,* then it can sometimes remind me to like get my own food and things like that.” (Kelsey*,* owner of two cats)*.

These participants seemed to find that an alignment between their schedules and their pets’ schedules was helpful with their personal recovery. Laura continues to discuss how caring for her dog’s needs extends to caring for her own needs, thus pet care encouraged one’s own self-care:

*“She [dog] has her breakfast at 8:30 in the morning. She has her dinner at 6:00 PM. She needs to be walked during the day. She wants treats and snacks and things during the day and being able to stick to that*,* it reminds me that*,* you know*,* this little dog*,* she needs it. So*,* me as a human*,* I also need it*,* so it motivates me. She’s having breakfast*,* I probably should have breakfast as well. And when she’s having dinner*,* well*,* I should probably start thinking about having dinner myself. So it’s almost a reminder to myself. I can use her as a parallel.” (Laura*,* dog owner)*.

Pets were also role models for many for building a healthier relationship with food:

*“I like to think*,* oh*,* like my dog doesn’t have like any negative connotations around food or around her body or anything. She’s not noticeable of these things. Like she more like if you give her a treat*,* she’s like so happy or like if you give her the whole bag of treats*,* she’s like over the moon.” (Gabriella*,* dog owner)*.

*“I observe my cats and the way they just eat when they’re hungry…I think that makes me want to be able to kind of respond to my body’s physical needs in a similar way.” (Kelsey*,* owner of two cats)*.

*“It sounds silly*,* but she [dog]*,* the way she doesn’t care what she eats*,* she never cares. I almost I look at her and I’m like*,* well*,* if she doesn’t care*,* why should I?” (Laura*,* dog owner)*.

These quotes illustrate the ways in which participants observed their pets’ healthy and non-judgmental relationships with food, and then considered how they could build their own heathier relationships with food and their bodies. This process may illustrate that participants’ recognition of the pets’ relationships with food enabled participants to potentially build greater self-compassion and decrease weight stigma.

#### Subtheme 1B: shaping healthy and unhealthy exercise patterns across recovery

Participants who experienced challenges related to compensatory exercise found that their pets, especially dogs, could be an “excuse” or a reason to engage in exercise to unhealthy extents. This was true especially at earlier stages of recovery:

*“He’s [dog] supposed to walk two hours a day*,* which has also a bit sort of made me exercise a bit more than I should. So it’s given me an excuse to exercise.” (Ben*,* dog owner)*.

*“It was an excuse to to go out and*,* you know*,* well*,* the dog needs to be run*,* you know*,* you know*,* the dog needs to be walked. And I thought*,* well*,* if the dog was running*,* why don’t we run?… It’s not negative because I’m helping the dog.” (Frances*,* owner of dog and cat)*.

*“It’s kind of bad because I can use her [dog] as an excuse to go out for more walks and longer walks because I’m like*,* I’m just walking the dog.” (Naomi*,* dog owner)*.

The challenges highlighted by Ben and Naomi provided insight into the challenges that individuals earlier in recovery may face related to dog ownership and managing recovery, while Frances, who appeared to be further along in her recovery, also retrospectively discussed a challenge that she had experienced earlier in her recovery journey. These participants highlighted that they exercised in likely unhealthy ways but were able to justify these behaviors as related to their dogs’ needs.

Participants also noted that their dogs played influential but differing roles in their exercise patterns at different times in their recovery journeys. Frances and Janine, who both owned dogs, felt that their dogs had previously contributed to an unhealthy relationship with exercise, but later helped to heal this relationship:

*“So I think to a certain degree*,* though that started negative*,* negatively because I could use her as an excuse for my exercise behaviors*,* it’s become a positive because there again*,* I now see that she [dog] moves intuitively and again if I feel like she wouldn’t push herself and I wouldn’t push her*,* I’m not gonna push myself.” (Frances*,* owner of dog and cat)*.

*“I think it really helped me…like if I’m in a bad headspace and wanted to get out*,* take them out*,* and then I just keep walking round and round and round and round. But I’d say that*,* it’s helped me to not do that. It’s helped me to kind of go on a walk to be with them [dogs]*,* play with them to be with them…I definitely notice I don’t take them out just to exercise*,* I take them out to play with them or to give them what they need… So it definitely did give me a different purpose which helped.” (Janine*,* owner of two dogs and four cats)*.

These participants both highlighted the importance of shifting focus from exercise to control shape and weight, to an opportunity to be mindful of their bodies and connect with their dogs. This shift in purpose and perspective shows how important the development of relational identity and compassion can be for personal recovery.

Cat owners, owners of dogs who needed less exercise, or owners of small dogs seemed to experience less of a negative impact from their pets related to compensatory exercise throughout their recovery journeys. For example, Paula had two dogs and a cat, and noted different experiences regarding compensatory exercise with pets based on pet type:

*“It seems like a really silly example, but especially when you know you have like a near compulsion to be always moving and doing stuff and exercising, when your kitten, who has been a velociraptor for about 12 solid months, goes, I’m going to curl up on your lap for an hour like a sweetheart, you go, actually, do I really need to get up and do those 10,000 extra steps? Maybe, maybe I’ll stay here.”*

However, she also said, *“I think the dogs definitely added pressure to be physically active.” (Paula*,* owner of two dogs and one cat)*.

These quotes from Paula highlight how different the impact of cat ownership versus dog ownership is related to exercise behaviors.

Similarly, Isabella, who had a small dog, noted that she was able to benefit from the companionship of her dog while she was on bed rest due to her eating disorder symptoms, without feeling guilty about being unable to walk her, due to her dog’s low exercise needs:

*“I suppose positive is just the fact that like she is a small dog and like I’m not having to take her on a walk like four times a day. So when I was kind of on like bed rest at home or I wasn’t really allowed to do much physical activity*,* I had her by me. Yeah*,* so I kind of didn’t feel like the guilt of maybe not taking her out or not doing this because I didn’t have to there.” (Isabella*,* dog owner)*.

This quote highlights that for this participant, not only was she not engaging in compensatory exercise influenced by her dog, but she also experienced comfort from her dog’s companionship without guilt related to not meeting her needs. Additionally, this highlights that dog owners experiencing eating disorders may face different vulnerabilities and protective factors depending on their dogs’ breeds, sizes, and exercise needs.

Participants who found their pets (both cats and dogs) to be helpful in preventing compensatory exercise felt that this occurred through several mechanisms. One mechanism was by being able to remain at rest with their pets instead of exercising:

*“I do think sometimes like my cat is*,* I don’t know*,* he’s quite good at sort of coming to see me and I guess like any behaviors*,* like exercise-related behaviors*,* it probably would help reduce that just because if he comes up to me*,* I’m going to sit with him.” (Anna*,* cat owner)*.

*“If it’s like if it’s a really horrible rainy day*,* like it can be in the UK sometimes and I’m kind of*,* I mean*,* it’s horrible outside*,* windy*,* like no one’s on a walk and I’m thinking*,* I need to get out*,* I need to do this*,* I need to do that*,* if she’s [dog] like curled upon the sofa just like chilling*,* I suppose*,* I suppose I feel more of kind of like*,* I’m just going to sit with her and we can just chill together and we can have like a nice day inside rather than I have to go outside and do this and do that.” (Isabella*,* dog owner)*.

These participants highlighted that sitting with their pets allowed them to give themselves permission to rest rather than engage in compensatory exercise behaviors, further emphasizing the importance of developing relational self-compassion.

A second mechanism by which pets helped participants not engage in compensatory exercise was by reframing movement from a mechanism to control one’s shape or weight to an opportunity for participants to connect with nature. For example, Caitlyn reported that walks became an opportunity for her to enjoy the scenery of her countryside environment, while Gabriella reported that she would not track her walking distance when walking her dog in the woods:

*“When we would be able to go on the quite long walks*,* he [dog] likes to sniff around and so it kind of is and whereas I’m like wanting to maybe pace*,* he’s wanting to actually enjoy it*,* so it could be frustrating. But equally I remember so many points just so I’d stop with him and be a little bit annoyed. But then you just look around and like I said*,* it’s kind of in the country. We might be around the beach or just in the country and I just think that kind of mindfulness of that I’m out*,* like*,* this is so beautiful*,* actually pay attention to what you’re doing.” (Caitlyn*,* owner of one dog as well as a dog and cat who passed away)*.

*“I find it quite different if I’m going*,* for example*,* for a walk by myself or if I’m taking my dog for a walk because I feel like if I if I’m like taking myself for a walk sometimes it can feel very like driven by the disorder and like*,* how many steps am I doing? Like how long am I walking for? Like all of this*,* like numbers*,* numbers. Whereas if I’m taking my dog for a walk*,* my phone’s in my pocket. I’m not tracking how far I’m walking. I’m more just going the woods*,* she likes the woods and like throwing the ball and like it doesn’t really matter how many steps I’ve done or like stuff like that. It’s more for the enjoyment.” (Gabriella*,* dog owner)*.

These quotes demonstrate dog walking as promoting a sense of mindfulness, and engagement with the present moment.

Similarly, Helen reported that playing with her cats helped her to become more in tune with her body:

*“Rather than just sort of sitting and wallowing*,* you’re up and about and you’re playing with them [cats] and it kind of makes you feel good. You’re moving your body. I think it gets you more kind of in tune with your body.” (Helen*,* owner of two cats)*.

This quote highlights a shift to mindfulness and kindness towards one’s body, potentially indicating the development of an increased awareness of interoceptive cues and increased body trust, through the human-pet relationship.

A third mechanism by which pets were helpful in preventing compensatory exercise was through participants developing a sense of mindfulness regarding their pets’ welfare such as physical limits:

*“With regards actually to kind of exercise*,* walking [DOG 2] as he was getting older*,* I was like*,* I can’t force*,* like OK*,* I might want to do more steps*,* but I can’t force him to*,* that’s not fair.” (Caitlyn*,* owner of one dog as well as a dog and cat who passed away)*.

*“I’m not going to force my animals to continue walking and walking and walking. So why should I do it to myself if that makes sense?” (Janine*,* owner of 2 dogs and 4 cats; referring to her dogs in this quote)*.

*“I will follow her [dog] lead on walks. When she tells me she’s had enough*,* you know*,* in her way*,* I will respect that and I won’t push her because I don’t*,* I don’t want to harm my dog. I love my dog so much.” (Laura*,* dog owner)*.

These quotes highlight participants’ acknowledgement of, and compassion for their pets’ physical limitations, and how they might apply this to reduce their own unhealthy exercise behaviors.

A fourth and final mechanism by which pets prevented compensatory exercise was by providing participants with other responsibilities, such as caring for their pets:

*“During the day I needed to look after him [dog] and I couldn’t just exercise all day*,* every day*,* when that’s really what I wanted to do.” (Ben*,* dog owner)*.

*“I know that I have to have time set out for her [cat] just for her own well-being and so her needs are tended to and just to get out of that cycle so*,* I know that I can’t give all of my time to behaviors.” (Quinn*,* cat owner)*.

These participants felt that caring for their pets took precedence over compensatory exercise.

#### Subtheme 1C: pets as mirrors - the person beyond the eating disorder

Participants highlighted key ways in which their pets had influenced their thought patterns, body image, and self-identity, such as providing unconditional acceptance and love regardless of the pet owner’s body shape or weight and providing a sense of identity beyond the eating disorder.

*“They [pets] offer that distraction*,* that focus*,* that unconditional love*,* you know*,* and I think certainly when it comes to sort of like body image and all of that*,* it’s like*,* they don’t give a crap what you look like.” (Caitlyn*,* owner of one dog as well as a dog and cat who passed away)*.

*“Your dog doesn’t care what you look like. She just cares about like your personality.” (Gabriella*,* dog owner)*.

*“She’s [dog’s] loved me at all sizes. She’s loved me when I’ve been at my smallest*,* my biggest and everything in between. So that’s definitely a positive I’ve seen in her. She*,* it doesn’t matter to her what I look like as long as I’m me.” (Laura*,* dog owner)*.

These participants felt that their pets cared for them based on their core selves, and not because of their body shapes and sizes, highlighting the importance of self-acceptance and self-compassion, and indicating the power of a relationship that feels free from objectification.

Some participants felt that their pets provided a link to a sense of identity outside of their eating disorders:

*“When I became ill*,* because I don’t know*,* I see disorders*,* they just put up a wall between you and everything else. And whereas he [cat] was kind of like he knew me before I was ill. So it was almost*,* I don’t know*,* it kind of enabled me to maybe access a bit of my child self that wasn’t eating disordered” (Caitlyn*,* owner of one dog as well as a dog and cat who passed away)*.*“One thing I always remember being told to do throughout services and inpatient and all this*,* you’d get a sheet and it’d be like*,* what are my likes and dislikes? And I had a point at one point where I was like*,* I genuinely don’t know because your sense of self becomes so linked in and tied in with your eating disorder*,* that you struggle to separate it. You don’t know who you are without it… Even if I didn’t know*,* just be*,* I like animals*,* I like my dog…so it’s nice to kind of start to build that sense of identity back and self-worth and nice to have that kind of identification.” (Isabella*,* dog owner)*.

*“I often get asked*,* like*,* what do you want to do with your life? Like what’s important to you? And I kind of freeze. I’m like*,* oh*,* I don’t know*,* but my go-to in the last few years or ever since I’ve had pets is always my animals.” (Janine*,* owner of 2 dogs and 4 cats)*.

These quotes demonstrated the ways in which disordered eating can constrict one’s sense of identity, and the ways in which pets have helped these participants expand their sense of self past their eating disorders in order to incorporate their pets, thus providing a stable and continued sense of identity as a pet owner and pet lover. The development of the relational identity of pet owner and pet/animal lover has important implications for ED illness processes, including anxiety and self-isolation. For individuals with EDs who are highly socially anxious and struggle to manage self-isolation, pet companionship may help to rebuild a positive identity.

#### Subtheme 1D: caring for pets, caring for the self - motivation to engage in recovery behaviors

Pets offered motivation to recover and to engage in recovery-oriented behaviors. Some participants noted that they were motivated to engage in recovery-oriented behaviors to better care for their pets both physically and emotionally.

Some participants were motivated by their pets’ physical needs, such as going for walks with their dogs, picking up their cats, and providing general affection to their pets:

*“On the days when I’ve been sort of gone back to restricting a bit and I’ve felt a bit weak towards the end of the day*,* I’ve then eaten something because I know that I’m gonna have to take him [dog] for a walk. So I know*,* ’cause then I’ve had to eat because I need some energy to take him.” (Ben*,* dog owner)*.

*“It’s kind of knowing that you have to keep yourself well to be able to look after them [cats]. Like I think I had a particularly bad time earlier this year where I was really quite acutely unwell and my one cat*,* I couldn’t. He’s quite a big cat and I actually couldn’t really pick him up because without getting really dizzy and lightheaded and I think that the guilt of him kind of pawing and wanting to be picked up and not really understanding why I couldn’t*,* I wasn’t doing that like I would normally. It’s almost kind of that external kind of wake up call to be like*,* you know you’ve got*,* you’ve got some creature relying on you to look after them” (Helen*,* owner of two cats)*.

*“I just think the motivational point like they’re a good push point to keep going because you have to be*,* you want to be well enough to look after them [pets] and and to be there for them and to be able to give them the quality of kind of affection and care and life that they deserve.” (Isabella*,* dog owner)*.

These quotes highlight the ways in which participants felt that their pets helped to enhance their recovery-oriented behaviors, in order to have the physical energy to provide care for their pets.

Other participants were motivated by being the person they would like to be as a pet owner, including having the emotional resources to care for their pets:

*“I know that I’m not the person that I want to be for them [pets] when the eating disorder is more dominant.” (Caitlyn*,* owner of one dog as well as a dog and cat who passed away)*.

*“When I’ve been really struggling*,* like with my eating disorder*,* and I know a lot of people have had the same experience*,* it can make you like*,* obviously not very nice person to be around because you’re just really irritable and stuff. And I’ve actually had this specific towards my dog*,* and I actually feel like when I was struggling the most with my eating disorder specifically is when my relationship with my dog wasn’t as strong and like when I’ve been better*,* it’s*,* she seemed to like me more now and like when I was better than in like during bad times ’cause I feel like I was a very irritable person and like grumpy.” (Gabriella*,* dog owner)*.

These participants highlighted that their pets also provided motivation to engage in recovery-oriented behaviors in order to have the emotional energy to provide care for their pets.

### Theme 2: pets and treatment pathways - the influence of companion animals on eating disorder recovery

*Summary*: Pets played a variety of roles in the treatment experiences of participants. Pets provided motivation to engage in treatment, and were sometimes involved in treatment through mechanisms such as providing support and being present. However, pets also occasionally posed barriers, especially to participating in inpatient treatment.

#### Subtheme 2A: paths to recovery - pets as catalysts for starting and sustaining treatment

Pets provided a reason for some participants to initially engage in treatment and increased motivation to get well. Helen and Paula reported that their pets were catalysts to seeking help:

*“Those initial GP calls were often*,* there was at least some part motivated by*,* you know*,* my*,* I want to make sure I can care for my cats and you know*,* I’m still around for them.” (Helen*,* owner of two cats)*.

*“I’ve really been struggling to do stuff*,* like my energy has been really low*,* I’ve not been feeling very well. So a lot of the stuff I was doing for them [pets]*,* like even just feeding them*,* taking them to classes*,* like my partner was picking up the slack and it was great because it meant they weren’t going without*,* don’t get me wrong. But again*,* that kind of made me feel like I should be the one able to do these things and I feel guilty and not good enough*,* but not having the energy and you know you did 20*,*000 steps today*,* but you couldn’t like take an hour out of your day for your dog. Like what kind of owner are you*,* kind of talk going on in your head. So I think that definitely was like a contributing factor of me starting the conversation with my psychiatrist about it.” (Paula*,* owner of two dogs and one cat)*.

The above quotes capture the negative emotions such as guilt and self-criticism that some participants experienced related to not feeling well enough to take care of their animals; this was a catalyst for change and increased motivation for initial help-seeking.

Pets also provided a reason for many participants specifically to complete inpatient treatment, in order to return home to be with their animals:

*“They’ve [pets] provided motivation to sort of keep going because*,* especially when I’ve been in hospital*,* I wanted to get back to see them and I think actually my - the rabbit that her sister that died*,* she actually died whilst I was in hospital. So I found that quite hard because I sort of I wasn’t there. So*,* but then it sort of motivated me to work really hard to get out because I wanted to then be there for my other rabbit.” (Anna*,* cat owner)*.

*“When I was inpatient*,* even if I didn’t want to do what they were telling me to do*,* it was like I’m going to do this so I can get home to her [dog]. So they can be kind of like a positive kind of push point when you know they’re waiting for you at home.” (Gabriella*,* dog owner)*.

These quotes highlight how pets and wanting to be reunited with them at home were often motivators for persisting with treatment continuation in inpatient settings.

For some participants, having a memento of their pets while inpatient provided additional and more tangible motivation to get well:

*“Every time that I’ve been inpatient*,* there have been*,* I’ve had pictures of them [pets] up on my scrapboards or whatever it is just as kind of reminders of kind of what I’m missing*,* but also what I’m*,* like why I need to stay and get well. So they’ve always featured heavily and been motivating.” (Caitlyn*,* owner of one dog as well as a dog and cat who passed away)*.

*“I know when like*,* like I was in hospital*,* I’ve got a pillow with her [cat] face on it and I had that with me when I was there. Yeah*,* so like people would often say like*,* oh like you need to eat this because*,* so you can get home and see your cat and stuff like that. And I was like not feeling very motivated. I was kind of like*,* I don’t really see the point in like carrying on. People would be like*,* you know*,* we’ve got like a beautiful cat at home and stuff.” (Mia*,* cat owner)*.

This theme highlights how physical reminders of pets can provide support and motivation, even when pets themselves are not physically present.

#### Subtheme 2B: pets as partners in treatment - how companion animals enhance therapy, build rapport, and support recovery

Participants involved their pets in their eating disorder treatment in a variety of ways. One way was through supporting them during telehealth appointments. Pets were able to help calm participants and provide emotional support during these appointments:

*“The positive thing has been sort of during difficult appointments with my dietitian because we do them over teams*,* he’s [dog] been here to keep me calmer.” (Ben*,* dog owner)*.

*“My [cat 3] would lay across my shoulder*,* so I always had them there to help me get through the Zoom calls*,* online therapy*,* like stuff like that.” (Janine*,* owner of 2 dogs and 4 cats)*.

These participants illustrated that pets provide support for online appointments through physical touch and presence.

Participants reported that they also sometimes received visits from their pets while they were inpatient, and found this to be a positive experience overall:

*“One of the first places that I was inpatient in was relatively local*,* so [dog 2] could actually come up and we got to walk him around the ground. So that was kind of lovely.” (Caitlyn*,* owner of one dog as well as a dog and cat who passed away)*.

*“Yeah. So I mean kind of when I was in inpatient*,* they bought her [dog] to visit me. So that was a nice thing to kind of like keep me going there.” (Isabella*,* dog owner)*.

These quotes highlight how supportive and helpful participants found their pets visits to be while inpatient.

Pets helped enhance rapport and relationships among patients with one another, and between hospital staff and patients as well, which improved the experience of treatment:

*“I think it can help the people around you as well because like I’d go upstairs and the other patients would be like*,* Oh my God*,* like we saw your dog on a leash outside. Like when’s it next coming? Like we’ll come out*,* we’ll see it. You weren’t allowed them on the ward with you*,* but it*,* be*,* it can be really nice just to kind of for other people to have that as well” (Isabella*,* dog owner)*.

*“I mean*,* there’s a girl I’m in treatment with and*,* I mean*,* I’ve been there seven-ish months now*,* nearly eight*,* and she’s been there a lot shorter than me. But we got to know each other really well by talking about our dogs because she has such a good relationship with her dog as well. And we’re planning on taking our dogs out soon together.” (Janine*,* owner of 2 dogs and 4 cats)*.

*“I was just about to bring up rapport and sort of bringing up*,* bringing up*,* breaking up that very clinical at times environment.” (Quinn*,* cat owner)*.

These quotes highlight the ways in which pets helped participants build a sense of community while inpatient, both with other patients, as well as with staff and clinicians.

In contrast, Naomi noted that when staff attempted to use visits from her dog as motivation, this damaged rapport in these therapeutic relationships:

*“They [inpatient staff] wouldn’t let me have a visit with her [dog]…when I was in the [inpatient facility]… They would sometimes be like*,* oh*,* you can only have like your mum and like [dog] visit if XYZ …* Just made me hate the team even more*” (Naomi*,* dog owner)*.

This participant highlighted that she felt that the ways in which hospital staff attempted to motivate her via her dog’s visits were harmful to her. She also discussed how this damaged her trust in and rapport with her treatment team, creating a strained environment in the treatment setting.

#### Subtheme 2C: the challenge of separation - how pet attachment creates barriers to treatment engagement

Pets also sometimes posed challenges and barriers related to engaging with treatment. Some participants shared that they had declined higher levels of care, or felt worried about accepting higher levels of care, such as inpatient treatment, due to concerns about their pets and not wanting to be separated from them, especially if a strong bond was present:

*“Luckily*,* I never had to go inpatient*,* but there was a time where we weren’t sure if I would have to and my concern was*,* but what will happen with [dog]? You know*,* who’s going to look after [dog]? And I’m very lucky. I’ve got family*,* I’ve got friends*,* I’ve got people around me that would have helped. But it was definitely something that I was worried about.” (Laura*,* dog owner)*.

*“It’s kind of maybe hard to imagine not having her there*,* but I did have like a couple of weeks ago*,* there was a question of maybe did I need to be admitted and I kind of felt like leaving in the morning saying goodbye to her [cat] as like*,* I really don’t want to be away from you again.” (Mia*,* cat owner)*.

*“I think I was always really worried about being sectioned…And I think a big part of that was like*,* obviously there’s loads of things you worry about in terms of losing freedom*,* but it was like I would not*,* like I*,* she [cat] would probably cope and she’d be OKish*,* but*,* yeah*,* just the thought of like not being around for her for any reason.” (Oscar*,* cat owner)*.

These quotes highlight barriers posed by pets to participants who potentially need high levels of care, due to their responsibilities of pet ownership and concern for pet welfare.

Missing pets while in inpatient was also a challenge for some participants, which made engaging with inpatient treatment difficult emotionally without the support of their animal:

*“I think maybe I missed my pets a lot when I was inpatient and that made it like even harder. I felt even more alone.” (Janine*,* owner of 2 dogs*,* 4 cats).*

*“It made me want to see her*,* you know*,* when you can’t*,* you always want what you can’t have more. It’s like it made me miss her [dog] a lot more knowing that I couldn’t like just go and see her.” (Naomi*,* dog owner)*.

These participants highlighted the struggle of being away from their pets while inpatient, amplifying loneliness and negative emotions.

Ben also experienced increased challenges related to engaging in outpatient treatment due to worries about leaving his dog home alone for too long:

*“To go to some of my appointments*,* the journey is 2 hours each way. So that would mean being out for five*,* maybe six hours because the bus only runs once an hour. So that would be difficult for both me and him [dog] because that is quite a long time for him to be on his own. So that has been a negative thing.” (Ben*,* dog owner)*.

This quote highlights the challenge of pet ownership related to being away from home for extended periods of time. Together, these quotes demonstrate that emotional bonds formed with pets, as well as their care needs, might be an additional challenge, or at least a consideration, for both inpatient and outpatient treatment.

## Discussion

The present work provides an in-depth exploration of the relationships that individuals with EDs have with their pets, and how their pets influence their eating and exercise behavioral patterns, affect regulation, self-identity, recovery motivations, and treatment experiences. These findings are novel, with the present work being the first published study to focus solely on the experiences of pet-owning individuals with EDs.

The present work revealed the complex interplay between stage of personal recovery, pet ownership, and the ways in which pets were helpful or created challenges related to participants’ ED-related behaviors. In early recovery stages, participants reported more ED-related challenges with their pets, in both eating and exercise behaviors. These challenges included pets’ needs conflicting with mealtimes, engaging in restrictive behaviors by giving away food to pets, and pets providing an “excuse” to over-exercise. However, later in recovery, participants generally seemed to find their pets much more supportive and helpful. Support provided by pets included co-eating meals thereby stabilizing eating patterns, pets modeling a healthy relationship with food and the body, and pets helping to both heal relationships with exercise and reduce compensatory exercise. These findings are particularly important when considered regarding key ED treatment goals, which include stabilization of eating patterns and reduction of compensatory exercise [27]. Notably, a mechanism by which pets were helpfully related to eating and exercise behaviors was via helping participants with affect regulation. Pets helped to lower the general level of stress and anxiety, which increased overall meal plan adherence. Additionally, pets helped participants manage distress before, during, and after meals, through providing support, physical touch, and distraction. This finding aligns with existing literature indicating that pets are associated with lower levels of distress and negative affect [18, 48]. Importantly, participants also reported increased mindfulness and attunement with their bodies, indicating a potential increased awareness of interoceptive cues and increased body trust through their relationships with their pets. This has important implications for personal recovery for individuals with EDs due to the links between impaired interoceptive functioning and low body trust in individuals with EDs [10, 15, 16]. Furthermore, participants identified that recognizing their pets’ healthy and non-judgmental relationships with food helped them to decrease judgement around food as well, potentially indicating an increase in self-compassion and a decrease in weight stigma [1, 25]. In sum, the present work revealed a complex relationship between the impact of pet ownership and stage of personal recovery.

Adding further nuance to this complex interplay is the role of pet type. Participants’ experiences were diverse and were impacted by whether they owned dogs or cats. Owners of dogs, especially large dogs needing higher amounts of exercise, more often reported engaging in compensatory exercise which was facilitated by attending to their pets’ needs. In contrast, owners of cats or dogs needing less exercise were less impacted by compensatory exercise in the context of pet ownership. However, owners of dogs needing high levels of exercise also reported that their dogs helped them to heal their relationships with exercise later in recovery, by reframing exercise from a mechanism to control shape and weight to an activity providing engagement with nature and being present and mindful. Existing literature indicates that dog ownership can be beneficial in general populations due to exercise facilitation [39, 46], but the findings of the present work indicate that this relationship is much more complex for individuals with EDs. However, for individuals in later stages of recovery, dog walking seemed to be helpful in healing participants’ relationships with exercise because dog-related exercise tends to be structured and centered around the animal’s needs, also providing connection to nature and an opportunity for mindfulness [17, 39, 63]. In sum, the present work highlighted a complex interplay among pet type and recovery stage impacting how beneficial participants found their pets to be.

Participants highlighted several ways in which their pets were helpful regarding their self-identity and thoughts about themselves. Importantly, participants felt that their pets provided unconditional love and acceptance, regardless of appearance which facilitated a positive self of sense and a continued sense of identity as a pet owner and pet lover. Unconditional love from pets has been previously explored, specifically within vulnerable populations, such as unhoused individuals and LGBTQ+ individuals, who experience increased social stigma [14, 20, 47]. The findings of the present work indicate that pets provide similar unconditional love to individuals with EDs, and importantly, provide a relationship absent of perceived judgement related to shape or weight. Additionally, pets provided participants with a sense of identity outside of their EDs, establishing a salient link to a non-disordered sense of self. Development of relational identity and relational self-compassion may be a helpful finding in the context of treatment and recovery [24, 49]. This may be a helpful finding in the context of treatment and recovery. A useful intervention for many individuals with EDs can be an exploration of other areas of life influencing self-identity (e.g., accomplishments at work, relationships with family and friends, engagement in hobbies and other interests [27]. The results of the present study indicate that pet ownership can also influence the sense of identity of individuals with eating disorders, helping them to expand their sense of self.

### Treatment implications

The present work reveals that pets may play an important role in recovery motivation and treatment experiences. Participants noted that a key motivation for recovery was to meet the physical and emotional needs of their pets, and that pets were both an important catalyst to start treatment and motivators to continue to engage in treatment. This aligns with existing work related to other types of recovery, including mental health, substance use disorder, and stroke recovery [34, 36, 41]. Some participants reported that having a memento of their pets, such as a photo, was motivating while an inpatient. This finding does not align with existing work indicating that viewing a picture of a pet does not lower stress [22]. However, the study by Ein et al., [22] was conducted in the context of conducting a brief arithmetic problem, and therefore these findings may not generalize to individuals separated from their pets for longer periods of time, as in the context of inpatient ED treatment. Some participants in the present work reported that they were able to receive visits from their pets while they were inpatient, and that this was helpful for their mental and emotional well-being while in treatment. Likewise, participants receiving outpatient treatment noted that pets were a calming presence during telehealth appointments. Pets did also pose challenges with some participants reporting that they would not want to engage in inpatient treatment due to concerns about leaving their pets; and with others who had engaged in inpatient treatment reporting that they missed their pets and that this caused emotional distress. Pets were therefore a barrier to help-seeking for some. This could indicate that more person-centered approaches are needed related to maintaining the human-pet bond, especially during higher levels of care for eating disorders.

### Limitations and future directions

All participants included in this study reported additional either diagnosed or suspected mental health concerns or neurodivergence, with common co-occurring diagnoses including depressive disorders, anxiety disorders, autism spectrum disorder, and attention-deficit/hyperactivity disorder. The interplay between EDs, co-occurring diagnoses, and pet ownership is complex and likely shaped by the experiences of individuals. Future research is needed to explore the role that these mental health concerns play in the experiences of pet owners with EDs. Further, participants who engaged in this study may have been more highly bonded to their pets than the general population, choosing to participate in the study because of their strong human-pet bonds. This is an important limitation because, although participants did discuss ways in which their pets posed challenges to recovery, participants’ strong bonds with their pets were often still reported as being beneficial to well-being. In contrast, individuals with poorer relationships with their pets may have endorsed additional negative impacts stemming from pet ownership. The study was limited to participants in the United Kingdom and because most participants were recruited from Reddit we may have recruited a relatively younger sample. Future studies should recruit a more diverse sample of participants in terms of age, location, and relationship experiences with pets. Additionally, although EDs commonly co-occur with autism, the rate of co-occurrence is estimated to be 4.7% [53]. In contrast, 58.8% of the participants included in our sample reported diagnoses or suspected diagnoses of autism (*n* = 10), potentially limiting the generalizability of this study.

The present work provides many directions for additional research. Future work could expand on the present study by including the experiences of pet owners of other types of animals, such as rodents, rabbits, reptiles, insects, fish, and birds. Studying a variety of human-pet relationships could provide unique insights into different relationship dynamics and models of care. Future research could explore including pets in the assessment and formulation process, in order to address ways in which pets may increase stress, but also facilitate strategies by which pets may be helpful. Future work could also operationalize some of the findings of the present study to explore whether interventions can be developed utilizing pets (e.g., whether spending time with pets before, during, and after meals can reduce restrictive or compensatory behaviors). This work could be useful in order to help individuals with EDs utilize their companion animals as additional support. Future research could explore the benefits of incorporating pets into inpatient settings or recommending that participants bring photographs/mementos of their pets. Further qualitative work as well as quantitative work may be beneficial to explore these experiences within a larger sample of individuals.

## Conclusions

Individuals with EDs experience complex relationships with their pets, with more challenges in earlier recovery stages, and more benefits in later recovery stages. Specific challenges include higher levels of compensatory exercise, restriction via sharing food with pets, and barriers to treatment due to concerns with separation from pets. Specific benefits include affect regulation, help with stabilizing eating patterns, healing relationships with exercise, clarification and expansion of the self-concept outside of ED-related identity, and treatment motivation. The present work provides a foundation for future research which can explore ways to mitigate challenges and amplify benefits of pet ownership. The present work also has relevance for the development of mental health treatment interventions for individuals with EDs.

## Data Availability

The data generated and analyzed during the current study are not publicly available due to participant privacy and ethical considerations but are available from the corresponding author on reasonable request.
